# The Educational Needs of Adolescent and Young Adult Renal Transplant Recipients—A Scoping Review

**DOI:** 10.3390/healthcare11040566

**Published:** 2023-02-14

**Authors:** Michael Corr, Clare McKeaveney, Fina Wurm, Aisling E. Courtney, Helen Noble

**Affiliations:** 1 Centre of Public Health, Institute of Clinical Sciences B, Royal Victoria Hospital, Belfast BT12 6BA, UK; 2 School of Nursing, Queen’s University Belfast, Belfast BT7 1NN, UK; 3 Regional Nephrology & Transplant Unit-Belfast Health and Social Care Trust, Belfast BT9 7ER, UK

**Keywords:** kidney transplant, adolescent, young adult, patient education

## Abstract

Renal transplantation is the gold-standard treatment for adolescents and young adults with end-stage renal disease. Despite enjoying excellent short-term outcomes, they suffer the worst rates of premature transplant function loss. Health behaviors: such as lack of adherence to immunosuppressive medications, are felt to be the major contributory factor. Understanding the educational needs of young renal transplant recipients allows healthcare practitioners to better support patients in managing their chronic disease. The aim of this scoping review was to understand what is known about their educational needs. A scoping review methodology was followed. Following an online search, study titles, and abstracts were screened for eligibility, followed by full-text assessment and data extraction. Data were qualitatively analyzed using thematic analysis. A total of 29 studies were included in the scoping review. In young people who struggled with self-management, three themes were identified (1) the Needs of the disrupted youth, (2) the Needs of the disorganized youth (3) the Needs of the distressed youth. There was a paucity of research to identify the protective factors that enable young recipients to successfully manage their health. This review outlines current knowledge of the patient education needs of young transplant recipients. It also highlights remaining research gaps that will need to be addressed with future research.

## 1. Introduction

Children on hemodialysis have a reported 55-fold increased mortality risk compared to the healthy population of a similar age [1]. Cardiovascular disease mortality accounts for 40% of deaths of young adult end-stage renal disease (ESRD) patients (500 times increased risk compared to the general population) [2]. Renal transplantation is the ‘gold-standard’ renal replacement therapy available to patients who reach ESRD providing significantly longer quantity and quality of life to patients [3]. Transplantation has been consistently shown to dramatically reduce mortality in patients with ESRD, with some studies reporting a reduction in up to 84% [4].

For young people, the benefits of transplantation are multiple and diverse and go beyond the excellent survival benefits. The overall accrual of disease burden is significantly less than when on dialysis, and a functioning transplant graft facilitates normal growth and development [5]. Young adult transplant recipients report increased exercise tolerability [6], better energy, and an enhanced sense of wellness compared to when receiving dialysis therapies [7]. Transplantation offers young people a less disrupted education and allows them to engage more fully in the workplace [8]. 

Despite the overwhelming benefits of transplantation, it is also associated with challenges. Many young transplant recipients (up to 30%) show symptoms in keeping with post-traumatic stress disorder (PTSD) after transplantation [9]. Patients can have body-confidence issues due to scars related to the operation [10]. Recipients also must become accustomed to attending regular appointments and adhering to a strict medication regimen whilst dealing with the complications associated with long-term immunosuppressive therapies (some with obvious early manifestations such as weight gain and acne seen with steroids) [11]. 

Adolescents and young adults enjoy better short-term outcomes following renal transplantation than any other age group [12]. Despite better initial outcomes, later rates of graft loss in adolescents and young adults are the highest of all age groups [13]. The mean survival of transplant grafts has been reported to be 7 years in younger recipients [14] compared to 10.4 years in all recipients from deceased donors [15]. This means that 50% of patients who have been transplanted in childhood will require a second transplant before reaching 25 years of age [16].

Albeit complex and multifactorial, the reason for the high rates of transplant graft loss in this group is often attributed to health behaviors and lack of adherence to prescribed therapies [17]. Adolescents and young adults have a higher rate of non-attendance to transplant appointments where monitoring and interventions to prolong graft function can be planned [18]. They also have higher rates of admission for transplant-related problems via emergency departments suggesting late presentation with complications—making recovery of transplant function more difficult [19]. Premature loss of a renal transplant is associated with increased mortality, increased morbidity, increased mental health problems, and decreased quality of life [10]. Finding a second suitable kidney for transplantation can often be difficult in this population due to the development of antibodies, and if compliance is suspected to be an issue, clinical teams can be wary of putting patients forward for subsequent transplantation [20]. Hence, young people with failed transplants often wait for long periods of time for a second transplant and are typically disproportionally represented in the suspended transplant waiting list [21]. 

Given the high association between health behaviors and patient education in young people with chronic diseases [22], it is crucial that healthcare practitioners are aware of and appreciate the educational needs of young transplant recipients. By understanding and ultimately addressing these educational needs, premature renal transplant loss in adolescent and young adult recipients may be avoided. The aim of this review was to understand what is known about the educational needs of adolescent and young adult renal transplant recipients within the literature. 

## 2. Methods

A scoping review is defined as “a form of knowledge synthesis that addresses an exploratory research question aimed at mapping key concepts, types of evidence, and gaps in research related to a defined area or field by systematically searching, selecting, and synthesizing existing knowledge” [23].

This study was completed adhering to the methodological recommendations and steps as laid out by Colquhoun et al., 2014, Levac et al., 2010, and Arksey, 2005 [23,24,25]. The six steps, as outlined by Arksey, 2005 were followed in the construction of the protocol, and any deviations were documented. The reporting of this review followed the reporting guidance by the PRISMA extension for scoping review [26], which can be viewed in the supplementary information. 

### 2.1. Eligibility Criteria 

All primary research related to adolescent (defined as aged 10–18) or young adult (defined as aged 18–24) renal transplant recipients’ educational needs were eligible for inclusion. Professional reports, quality improvement projects, and editorials were not included. Studies where other solid organ recipients were included and data pertaining to renal transplant recipients only could not be separated were excluded. 

### 2.2. Search Strategy 

From the research question, a search strategy was developed with the assistance of a medical librarian. Six online databases were searched for relevant articles-OVID-Medline, EMBASE, Web of Science, Scopus, CINAHL, and Psychinfo from inception until 17 December 2022. A targeted grey literature search was also conducted for further articles for inclusion. An example search strategy can be viewed in the supplementary information. 

### 2.3. Study Selection and Data Extraction

Initially, titles and abstracts of selected articles were reviewed for appropriateness for full text review against the inclusion of material by two members of the research team. The full-text review was then conducted independently by two researchers. Disagreements for full-text inclusion/exclusion were solved by consensus or arbitration by a third party. 

Data were then extracted from each included article using a pre-designed data extraction tool. A completed data extraction tool can be viewed in the Appendix A.

The following information was extracted from each selected study;

- Study characteristics: author, year of publication, country, design, sample size, clinical setting, number studied
- Population characteristics: adolescent vs. young adult, transplant function
- Study design: methods used to investigate educational needs
- Key findings

### 2.4. Data Analysis

Both qualitative and quantitative studies were identified, so a mixed methods approach was taken for data synthesis. The approach outlined by the Joanna Briggs Institute for mixed-method data analysis in synthesis reviews was utilized [27]. A convergent integrated approach was applied where quantitative data were initially extracted and then transformed into qualitative data by creating textual descriptions to create qualitative summaries of the data from the quantitative results [27]. These newly formed qualitative data were then coded by members of the research team along with the already qualitative studies using traditional content analysis [28]. In keeping with the scoping review methodology, data quality assessment nor assessment of bias for individual studies was not completed. 

The results of the thematic analysis were discussed in a consultative exercise with young transplant recipients and healthcare professionals involved in transplant education to enrichen and validate the analysis. This step aided in refined and bringing together individual codes to form the themes derived in the review and bring the patient perspective to our findings.

## 3. Results

**Figure 1:**
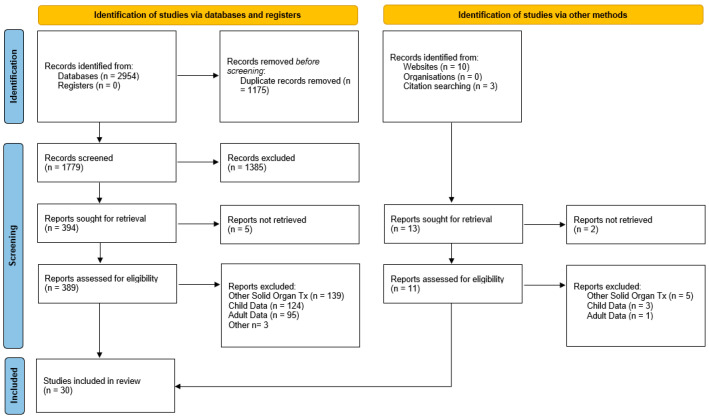
PRISMA 2020 Flow Chart.

A total of 2954 records were identified through database searching, and an additional 13 records were identified through grey literature searching. Following duplicate removal and abstract screening, the full text of 394 articles was reviewed. A PRISMA 2020 flow diagram [29] can be reviewed in Figure 1.

### 3.1. Qualitative Analysis of Selected Studies

Three themes of an investigation by researchers emerged from the studies following the thematic coding of the data:

1. The needs of the disrupted youth
2. The needs of the disorganized youth
3. The needs of the distressed youth

### 3.2. The Needs of the Disrupted Youth

Some young transplant recipients appear to face substantial delays in their education, and their levels of qualifications are lower than their peers [30]. The disruption to educational attainment is particularly marked by already vulnerable groups, such as those from ethnic minorities or those belonging to lower socioeconomic groups [8]. Those who do achieve high educational and professional attainment are more adherent to immunosuppression medications and are at a reduced risk of developing depression or anxiety [8].

Studies also investigated how disruption to their youth impacts young transplant recipients meeting major psychosocial milestones [30]. Young adult transplant recipients remain living with their parents at much higher rates than their peers and appear to be delayed in developing romantic relationships [31]. The delay in reaching psychosocial milestones in young transplant recipients reduced their sense of autonomy, their satisfaction with life, and their own attitudes to health [32].

Researchers suggest that young people who are supported to reach their full potential (either professionally or personally) seem to enjoy additional protection from anxiety and depression and even have better clinical outcomes, such as improved transplant function [33].

### 3.3. The Needs of the Disorganised Youth

The selected studies focusing on the importance of young transplant recipients developing organizational skills, engaging with their health, and having self-motivation as they hypothesized that this would lead to better transplant and overall health outcomes.

The largest reported barrier to developing self-reliance skills in recipients was a lack of knowledge and understanding of their condition and medications. Studies described how recipients feel disengaged with their own health and often are disempowered due to a lack of health knowledge [34]. Those who were transplanted at a young age were at the most risk of having insufficient health knowledge as education efforts at the time of transplantation were directed at their parents [35]. When recipients begin to take increasing responsibility for managing their own health, they often feel overwhelmed and underprepared and are subsequently more likely to partake in health-risky behaviors [36].

Many young adult transplant recipients lack coping mechanisms or strategies to help them deal with the extra organizational burden of being a transplant patient [31]. Researchers investigated barriers to the normal development of these important traits and skills in young transplant recipients. A lack of, or disruption to, the routine was suggested to be detrimental to recipients’ organizational skills acquirement. Periods such as weekends, leaving the family home, or transitioning from pediatric to adult health services were associated with reduced compliance to medication and were highlighted as times when support was most required [37]. Good family support and help in developing organizational skills, particularly with taking medications, were protective against poor health outcomes [38,39].

This transition from dependent child to independent adult often leaves recipients feeling unprepared, uninformed, and unable to become fully responsible for their health—increasing their risk of complications, especially around clinic attendance, blood sampling, and medication compliance [40].

### 3.4. The Needs of the Distressed Youth

Studies showed that young transplant recipients craved “normality” and wished to be viewed as similar to their peers [35]. Some young recipients had a level of disappointment following transplantation as they still did not feel “normal” and were unprepared for those emotions following transplantation [38]. Body image issues were commonly investigated by researchers [14], as were the side effects of medications related to transplants [41]. Young transplant recipients often reported high levels of depression and anxiety related to physical signs/symptoms related to transplantation and poor coping mechanisms to deal with this psychological burden [34].

Studies consistently demonstrated recipients reporting symptoms or diagnostic signs of anxiety, depression, irrational anger, and even post-traumatic stress disorder [39]. Psychological distress or a mental health diagnosis was the most important independent factor in predicting not only the quality of life of young recipients but also transplant-related health outcomes, such as graft survival [42]. Researchers found that young transplant recipients often have inadequate resilience or coping skills related to mental health [43]. The most deployed strategies observed in these young recipients were denial and avoidance (not wishing to talk about transplant/voice concerns). It was perceived that many young recipients had been inadequately prepared for the mental stresses associated with transplantation, were ill-equipped to be resilient to psychological stressors, and lacked sufficient support when dealing with mental health problems [44]. Two studies reported young transplant recipients who have developed symptoms consistent with PTSD. Interestingly PTSD symptoms did not seem to be related to clinical factors (such as the severity of illness, time on dialysis, and risk of mortality) but related to a complex adjustment to the variety of subjective stressors (body image, lacking normality, childhood illness, family dynamics) faced by young recipients [45].

## 4. Discussion

This review demonstrates the wide range of educational needs of young transplant recipients studied in the literature. Areas such as health literacy, self-management, and organizational skills may appear obvious and have been documented in studies of young people with other chronic physical illnesses [22]. Despite formal transition programs designed to aid this, some adolescent and young adult recipients still feel unprepared to manage their own healthcare and struggle when transitioning from pediatric to adult services [36,46,47,48,49,50,51].

However, educational needs go beyond the recipient’s understanding of their condition and how to take medications. The findings of this review would suggest that young recipients need additional support to meet educational/professional/developmental goals that are disrupted compared to their peers. Young people with other physical health conditions, such as diabetes mellitus [45], childhood cancer [46], and cystic fibrosis [47], have been shown to experience similar disruption. The inclusion of youth workers in the clinical team can help in advocating for patients and educating recipients and their families about the support available [22,43].

The review raises questions about how young people are prepared psychologically for transplant. Poor coping skills, disappointment from high expectations of life post-transplantation, and psychological toll all appear to be areas of educational need for young recipients. Given the associative links between mental health outcomes and overall transplant function [10,52,53,54], assisting young people in developing good mental health practices may be a ‘blind spot’ in educational need in pre- and post-transplant care.

Interestingly very few studies investigated protective factors or what allowed some young transplant recipients to be successful in managing the burdens associated with transplantation. From the search strategy, there were no papers that fully explored demographic, social, or psychological factors that were associated with good outcomes (either transplant survival or quality of life-related). There also appeared to be few studies that explored the lived experiences of young transplant recipients who managed their condition well without complications.

This review highlights potential areas of research to better understand the educational needs of adolescent and young adult renal transplant recipients:

(1) There is a paucity of research on how certain young people are successful and cope/adapt following transplantation which may help in developing tools to help other patients.
(2) Educational needs of racial, ethnic, and religious minority groups and those from immigrant or socially deprived backgrounds need further exploration as these may be different from the general population.
(3) An in-depth qualitative analysis of young transplant recipients, which fully explores the contextual factors such as healthcare setting, background, and resources, may help further our understanding of their experiences and requirements.
(4) Future studies that are designed and carried out with adolescent or young adult transplant recipients as equal partners may improve the quality and validity of the results.

### 4.1. Limitations

A scoping review is intended to map out and organize the current literature and does not assess the quality of the studies identified. Hence, the review is limited in its ability to confirm the validity of each study’s claim of young recipients’ experiences. However, given the wide-ranging and complex factors that affect educational needs, a positivist review methodology i.e., searching for the ‘right’ answer with a systematic qualitative review, would likely have also been limited in its conclusions. Truthfully, it will be hard for any review to generalize the experiences of adolescent and young adult transplant recipients from varying cultural and social backgrounds and healthcare settings around the globe. Therefore, the authors hope this review, inkeeping with scoping review methodology, will offer an appropriate understanding of the current literature and identify potential research gaps.

### 4.2. Implications

Support systems and health services need to be designed by clinicians to aid young people, and physicians should have knowledge of other professional services and members of the multidisciplinary team that can assist. Transplant physicians need to be particularly aware of the increased risk of anxiety, depression, and PTSD in these patients and be able to appropriately refer them to other services. Ultimately it is hoped that this review will aid in either targeting research or changing healthcare provision for young transplant recipients to improve their care. It should also highlight the importance of this issue and encourage more service providers to reach out and seek young recipients’ opinions and voices. The findings of this review may be of interest to patient advocacy groups.

## 5. Conclusions

This scoping review has outlined the literature that addresses the educational needs of younger transplant recipients and highlights the gaps in our knowledge. Hence, for clinical teams to develop best practices in the management of young renal transplant recipients, further research is required. The transplant community needs to prioritize good quality research that is patient-centred and patient-led to better inform practice. In-depth qualitative studies that seek to fully explore the lived experiences of young transplant recipients could provide better insights into their unique educational needs. This, in-turn, could help inform future intervention trials and hopefully improve outcomes for young recipients.

## Data Availability

All data related to this study can be found in the main manuscript or in the Supplementary Files.

## Supplementary Materials

The following supporting information can be downloaded at: https://www.mdpi.com/article/10.3390/healthcare11040566/s1, Table S1: Preferred Reporting Items for Systematic reviews and Meta-Analyses extension for Scoping Reviews (PRISMA-ScR) Checklist [26].; Table S2: MEDLINE & EMBASE search strategy.

## Appendix A

**Authors (Reference),** **Country**	**Aim of Study**	**Number of Participants**	**Adolescents or Young Adults**	**Study Design**	**Length of Study**	**Methodology of Assessment**	**Key Findings**	**Limitations**
Aasebø [30] Norway	Describe the life situation, lifestyle, and common activities of daily life in young adult kidney transplant recipients aged 18–35 years. In addition, compared their HRQoL with a general population sample, adjusting for age, sex, and education.	131	Both	Cross sectional study. Questionnaire. Short Form 36 (SF-36). Comparison made with matched general population.	Retrospective-snapshot	Short Form 36 (SF-36) questionnaire. compared groups using a two-sample t-test or Fisher’s exact test, or the Mann–Whitney U-test.	Recipients reported high participation rates in cultural and sports activities. Majority were working and satisfied. 25% of the total group were not integrated in professional life. The transplant recipients scored lower than the general population on seven of the eight SF-36 scales and the two summary scales after adjusting for age, sex and education. 70% stated had delayed education. 6% were happy with physical appearance-77% reported-ve change since transplant. Higher pain scores than general population.	Self-reported Questionnaire. 47% return rate. Predominance for females to return. Data of clinical status and transplant function not collected.
Akchurin [48] USA	Was there a difference in immunosuppression adherence in groups transitioning to adult services?	27	Adolescents	Retrospective Case control study	2 years	Measured tacrolimus levels. Statistical analyses including *t*-Test and multivariate analyses.	Transition not associated with changes in medication adherence.	Tacrolimus levels less than ideal to measure adherence. single centre didn’t cover those lost to follow-up
Anthony [11] Canada	To measure quality of life in adolescent transplant recipients and impact on their families.	12	Adolescents	Retrospective pilot study. Case control. Questionnaire.	3 years post transplant. Single study,	VAQOL and General Health, the PedsQL 4.0, PedsQL End Stage Renal Disease Module, and Impact on Family Module	High level of fatigue. Concerns about physical appearance. Reported difficulties in interactions with peer and family. Despite good outcomes family reported negative emotional outcomes, stress and worry. Education delay. Turbulent relationships both peer and family.	Small study. Self reported.
Ashoor [49] USA	To assess the prevalence and types of sexually transmitted infections in paediatric renal transplant patients	49	Adolescents and young adults.	Retrospective cohort observational study.	4 years	Observation	15% of men sexually active. 45% of women. 75% off sexually active women on contraception. 36% sexually active had at least one STI. Most patients rely on nephrologist for preventive care -important to raise awareness of this issue in the transplant community	Small study. Self reporting by patients,
Boucquemont [37] Canada/USA	If day of the week affected teen or young adult adherence to medications	138	Both	Post-hoc analysis of a prospective randomised control trial	15 months	Logistic regression with generalized estimating equations to estimate the association between week- ends/weekdays and each of perfect taking.	Weekends are disruptive to normal routine and patients have reduced adherence to medication at the weekends.	Post-hoc analysis. Small sample.
Bullington [50] USA	Understand opinions of adolescent transplant patients on why not taking medications	12	A	Qualitative	One interview and follow-up	Q-methodology	Three themes- medication issues. Deliberate non-adherence and “troubled adherence”.	Self-reported. Small sample.
Chaturvedi [51] Australia	Assess graft stability and patient satisfaction after transition from children to adult services.	11	Both	Cohort observational. Retrospective review of clinical notes	Over course of two year	Patient evaluations.	1 acute episode of antibody mediated rejection. Inadequate involvement of young people in transition planning. Lack of preparation for transition to adult services.	Small numbers, self reported.
Dobbels [10] Netherlands	assess HRQOL, depressive symptoms, side effect experience and treatment adherence in a sample of adolescent kidney transplant patients, using self-report and parent-report.	26	A	Cross-sectional study	Single episode	Self-reported questionnaires. KIDSCREEN-27 (QOL), a treatment adherence interview, the MTSOSD-59R (side effects) and the Beck Depression Inventory (depression)	Patients reported good QOL. Depressive symptoms occurred in 17.4%, and 75% were non-adherent with their medications. Many show problematic health behaviours. Side effects were increased appetite, fatigue and headache; the most distressing ones were hair loss or thinning of hair, warts on hands or feet, and sores in the mouth or on the lips.	Small. Cross-sectional design.
Hamilton [32] UK	wellbeing and medication adherence are associated with psychosocial factors using data from the Surveying Patients Experiencing Young Adult Kidney Failure (SPEAK) study	417	Both	Survey	Once off survey	multivariable linear regression to examine psychosocial associations with scores on the Warwick–Edinburgh Mental Wellbeing Scale and the eight-item Morisky Medication Adherence Scale.	Wellbeing was positively associated with extraversion, openness, independence, and social support, and negatively associated with neuroticism, negative body image, stigma, psychologic morbidity, and dialysis. Higher medication adherence was associated with living with parents, conscientiousness, physician access satisfaction, patient activation, age, and male sex, and lower adherence was associated with comorbidity, dialysis, education, ethnicity, and psychologic morbidity.	Cross-sectional design limits changes on outcome can not be tracked, directionability between variables could not be assessed.
Jakubowska-Winecka [38] Poland	To determine parental attitudes affect on adolescent medication adherence	197	A	Survey	Once off	M. Plopa’s Parental Attitudes Scale, which distinguishes 5 types of attitudes. Medication adherence was evaluated on the basis of the Morisky Medication Adherence Scale (MMAS-8)	Accepting attitude and overly protective attitude corresponded with increased adherence.	Other factors not controlled in study.
Kärrfelt [43] Sweden	To understand the emotional and psychological adaption of patients after undergoing transplant	20	A	Mixed methods interviews. Quantitative and qualitative interviews.	Once	Thematic analysis	Mostly felt unaffected. Improved relationship with donor. One deceased donor recipient had nightmares about alien kidney. Psychological adaption seemed to rely of denial & avoidance.	High drop out rate. Voluntary recruitment may have led to a selection bias.
Kim [52] South Korea	To understand the experiences of adolescents undergoing renal transplant	9	A	Qualitative descriptive study.	Once	Content analysis	“being different from others,” “not being invited as a decision maker,” “becoming one of them,” “still being different from others,” “having mixed feeling toward mothers,” and “coping with new circumstances.”	Small-specific population
Korus [40] Canada	Understanding the information needs of adolescent transplant recipients.	8	A	Qualitative descriptive study	Once	Content analysis	Transplant a stressful situation. 4 main stressors: changes in body image, wanting to be normal, pain, and breakdown in communication processes. Two coping strategies were gaining more information and seeking social support.	Small study
Kullgren [53] USA	Validate measurement tool “transplant responsibility questionnaire.” And determine between TRQ and adherence	59	A	Survey-TRQ scores vs TAC levels	Once	Bivariate correlations were calculated between TRQ average scores, caregiver–child TRQ discrepancy scores, and adherence. Oneway ANOVAs were used to assess differences between adherent and non-adherent groups on the TRQ	Adherence unrelated to TRQ score. Disparity between parent and recipient perception of self-management. Older more self reliant than younger	Self reported
Lugasi [33] Canada	To assess the identity formation of renal transplant patients and type 1 diabetic patients	85	A	Qualitative	Once	Demographic questionnaire. Quality of Life Profile Adolescent Version (QOLPAV)	Differences in ideological identity, with tx recipeints showing higher levels of diffusion and controls showing higher levels of foreclosure. No differences with respect to interpersonal identity, QOL, perceived control over the QOL domains, and perceived opportunities for growth and development were found	Convenience sampling. Questionnaire based data collection.
Malekahmadi [42] Iran	To evaluate the extent to which socioeconomic, clinical, and psychological characteristics explain the variance in the health-related quality of life of adolescent Iranian kidney transplant recipients.	55	A	Cross-sectional study	Once	Hierarchal regression analysis, the cross-sectional socioeconomic, clinical and psychological variables associated with health outcomes.	The relative predictive power of socioeconomic, clinical, and psychological variables with respect to health-related quality of life was 21.8% (*p* = 0.088), 21.2% (*p* = 0.014), and 27.6% (*p* = 0.001). Psychological factors had a greater relative predictive power in postrenal transplant health-related quality of life of adolescents than did the socioeconomic and clinical characteristics.	Small sample size. Cross-sectional rather than longitudinal analysis.
Massey [31] Netherlands	The aim of this study was to investigate (a) the extent to which age at first renal replacement therapy, achievement of developmental milestones, satisfaction of psychological needs, and coping were related to subjective well-being and medication adherence	62	YA	Cross-sectional interview study	Once	subjective well-being (Positive And Negative Affect Schedule; Satisfaction With Life Scale), medication adherence (Basel Assessment of Adherence to Immunosuppressive Medication Scale), dispositional coping (Brief COPE), achievement of developmental milestones (Course of Life Questionnaire), and satisfaction of psychological needs (Basic Psychological Needs Scale)	Sixty-five percent were classified as nonadherent in the past month. In contrast, subjective self-rated overall adherence was high. None of the variables measured were related to nonadherence. Higher feelings of competence and autonomy, and timely achievement of social and psychosexual developmental milestones were related to higher subjective well-being. Well-being and adherence did not differ according to age at diagnosis or first renal replacement therapy	Limited by cross-sectional and retrospective analysis. Self-selected participants so selection bias may be present. Small sample analysis.
Mellerio, [8] France	To document the semiprofessional outcomes of adults who underwent kidney transplantation before age 16 years between 1985 and 2002	374	A	Retrospective cohort study.	Once	Questionnaire which was then compared to data from the general French population.	The median ages were 27.1 years at survey time and 12.3 years at first transplantation. Of the participants, 31.1% lived with a partner (vs. 52.2%; PG0.01) and 35.7% lived with their parents.	Self-reporting-more frequently women and those with better graft function.
Mintzer [44] USA	To assess prevalence and correlates of self-reported symptoms of posttraumatic stress in a nonreferred sample of adolescent liver, heart, and kidney transplant recipients.	104	Both	Cross-sectional analysis	once	PTSD Index for the Diagnostic and Statistical Manual of Mental Disorders	16% of the adolescents met all symptom criteria for PTSD, and an additional 14.4% met 2 of 3 symptom-cluster criteria. Regression analysis indicated no effect of gender, ethnicity, age at interview, organ type, time since transplant, or age at transplant	Retrospective analysis. Patients transplanted over a large time span 80s to 90s.
Nguyen [55] Canada & USA	To gather the perspectives of recipients, parents, and health professionals concerning their needs, challenges, and potential intervention strategies to design an optimal, multi-component medication adherence intervention	32	Both	Qualitative study design- focus groups	Once	Content analysis-leading to themes	Multi-component behavioural intervention, including an expanded electronic pillbox and companion website, education materials, and customized digitized features to support shared responsibility and communication among recipients, parents, and health professionals were all suggested by participants.	Self-selecting so potential selection bias. Predominantly white population.
Penkower [39] USA	(a) describes the prevalence of psychological distress, (b) describes the prevalence of nonadherence, and (c) explores the association between the recipient’s psychological distress and his/her subsequent medical adherence	22	A	Qualitative design- interviews.	Twice	Beck Depression Inventory II (BDI). State Anxiety subscale of the Spielberger State-Trait Anxiety Scale. State Anger subscale of the Spielberger State-Trait Anger Scale.	At the initial interview, 36.4% had symptoms of depression, 36.4% endorsed anxiety, and 18.2% endorsed excessive state anger. Non-adherence rates were 13.6% for medication, 22.7% for blood work, and 50% for missed clinic.	Small study-pilot. Self-reporting adherence.
Quast [56] USA	The current study examines associations between personality (i.e., agreeableness, conscientiousness, neuroticism) and adherence barriers in a group of adolescent and young adult (AYA) solid organ transplant recipients	90	Both	Cross-sectional study	Once	Agreeableness, Conscientiousness, and Neuroticism scales from the NEO Five-Factor Inventory and the Adolescent Medication Barriers Scale (AMBS)	Lower levels of agreeableness and conscientiousness and higher levels of neuroticism were related to higher self-reported barrier scores (AMBS; r’s ¼ 0.31–0.53, *p*’s < 0.001). The relations differed by personality factor and barrier type.	Small study, self reporting-limited by cross sectional and retrospective nature of study design.
Quinn [36] USA	Explored the novel role of resilience constructs as protective factors in securing stable HCT among AYA with KT	32 (17 stable, 15 unstable).	Both	Qualitative study	Once	Semi-structured interviews. Content analysis.	Confidence in and connection to one’s healthcare team appear to be linked with a stable HCT among AYA with KT. This suggests that interdependence, the ability to foster connections with and elicit support from healthcare providers, as opposed to complete independence or autonomy, which is often advised in the HCT process, is a critical component of resilience linked to stable HCT.	Small study. Retrospective and clinical factor determining stable vs unstable less clear.
Simons [34] USA	To evaluate whether different factors would be associated with lower mental health scores on the CHQ	39	Adolescents	Comparative study	Once	Semi-structured interviews. Multiple validated questionnaires around medication, knowledge, mental health. Hierarchal regression analyses to determine strength of association.	Perceived frequency of medication side-effects and family conflict significantly contributed to adolescent physical functioning and mental health outcomes. Taken together, transplant consequences and family environment significantly impact physical and mental health outcomes in adolescent transplant recipients	Risk of type 1 error as multiple variables investigated.
Silva [54] Brazil	To assess the prevalence and correlates of nonadherence to immunosuppressive medications in a pediatric kidney transplant population who received free access to immunosuppressive medications within the health care system	156	Adolescents	Single centre cross sectional analysis	Once	Implementation nonadherence to immunosuppressive medications was measured by the 4 questions of the Basel Assessment of Adherence to Immunosuppressive Medications Scale. Multilevel correlates to non—adherence (patient, micro, and macro levels) were assessed	33% were nonadherent to immuno—suppressive medications, mainly in timing (25%) and taking (10.9%) dimensions. Being an adolescent (odds ratio: 2.66; CI, 1.02–6.96), religion other than Catholic or Protestant (odds ratio: 4.33; CI, 1.13–16.67), and family income higher than 4 reference wages (odds ratio: 3.50; CI, 1.14–10.75) were factors associated with nonadherence.	Convenience sample from a single centre. Adherence self-reported. Limitations associated with cross sectional design.
Tielen, Mirjam [57] Netherlands	To identify young people at risk of non-adherent behaviour.	26	Young adults	Comparative study. Q-methodological study.	Once	Questionnaire Q-methodology	Four distinct attitude profiles concerning posttransplant health lifestyle were found among these young adults: (a) concerned and controlled, (b) appearance orientated, (c) opinionated and independent, and (d) easy going and pliable	Self-selecting population. Pilot study.
Tong [35] Australia	To explore experiences and perspectives of adolescent kidney transplant recipients following kidney transplantation	22	Adolescents	Qualitative study	Once	In-depth interviews. Grounded and thematic analysis.	The overarching theme was achieving a sense of normality. Having the same opportunities and potential to achieve as other adolescents facilitated better adjustment, well-being and positive development after transplant.	Variability in some interviews parents present. Wide age range.
Varnell [58] USA	Assess barriers to taking medication for adolescents and young adults	98	Both	Prospective cohort study	Over two years	Patients assessed for 14 barriers to medication adherence using the barriers assessment tool	Patients with an identified barrier to adherence were more likely to have BPAR (*p* = 0.02) than patients without an identified barrier in the 24-months following barriers assessment.	Single centre
Wolf [7] USA	To quantify physical activity and grip strength in pediatric kidney transplant recipients and describe attitudes about exercise and exercise counseling given concerns about allograft injury	101	Both	Cross sectional analysis.	Once	Patients completed the Physical Activity Questionnaire (PAQ). Grip strength was measured with a dynamometer. We asked about activity limitations and provider counseling. Univariate analysis and multiple linear regression were used to determine independent predictors of PAQ score and grip strength z score.	Median PAQ score was 2.2 (range 0–5) and was lower compared with controls (*p* < 0.001). The average grip strength z score was −1.1 and −0.7 in the right and left hand, respectively. Predictors of lower grip strength were younger age (*p* = 0.036), non-African American race (*p* = 0.029), lower height z score (*p* = 0.010), and longer percentage of lifetime with kidney disease (*p* = 0.029).	Non-longitudinal design. Single-centre. Relying on patient recall.
Zelikovsky [59] USA	To examine the potentially modifiable barriers related to adherence among adolescent kidney transplant candidates	56	Adolescents	Cross-sectional study	Once.	Interviews- around medical adherence and semi-structured interviews (parents present). Medical Adherence Measure (MAM) adherence interview. Qualitative Study.	Better knowledge of the medication regimen was associated with fewer missed doses Patients who perceived more barriers had more missed doses. Patients who endorsed “just forget,” the most common barrier (56.4%), reported significantly more missed doses.	Patient self-reporting. Small sample size. Didn’t look at health provider related issues to adherence.
